# Potencies of Cocaine Methiodide on Major Cocaine Targets in Mice

**DOI:** 10.1371/journal.pone.0007578

**Published:** 2009-10-26

**Authors:** Erik R. Hill, Jinbin Tian, Michael R. Tilley, Michael X. Zhu, Howard H. Gu

**Affiliations:** 1 Ohio State Biochemistry Program, The Ohio State University, Columbus, Ohio, United States of America; 2 Department of Neuroscience and Center for Molecular Neurobiology, The Ohio State University, Columbus, Ohio, United States of America; 3 Department of Pharmacology, The Ohio State University, Columbus, Ohio, United States of America; 4 Department of Psychiatry, College of Medicine, The Ohio State University, Columbus, Ohio, United States of America; 5 Department of Biology, Central Methodist University, Fayette, Missouri, United States of America; INSERM U862, France

## Abstract

Cocaine methiodide (CM), a charged cocaine analog, cannot pass the blood brain barrier. It has been assumed the effects of systemic CM represent cocaine actions in peripheral tissues. However, the IC_50_ values of CM have not been clearly determined for the major cocaine targets: dopamine, norepinephrine, and serotonin transporters, and sodium channels. Using cells transfected with individual transporters from mice and synaptosomes from mouse striatum tissues, we observed that the inhibition IC_50_ values for monoamine uptake by CM were 31-fold to 184-fold higher compared to cocaine at each of the transporters. In dorsal root ganglion neurons, cocaine inhibited sodium channels with an apparent IC_50_ of 75 µM, while CM showed no observable effect at concentrations up to 3 mM. These results indicate that an equal dose of CM will not produce an equivalent peripheral effect of cocaine.

## Introduction

Cocaine produces complex behavioral and physiological effects including: addiction and locomotor stimulation, cardiac arrhythmias, and hormonal changes [1], [2]. The high affinity targets of cocaine include the dopamine (DA) transporters (DAT), norepinephrine (NE) transporters (NET), and serotonin transporters (SERT) [3]. Cocaine inhibits these transporters with similar potencies at micromolar or submicromolar levels [4].

Cocaine produces effects in the central nervous system (CNS) primarily by inhibiting three monoamine transporters, DAT, NET and SERT [3]. These transporters clear neurotransmitters from neural synapses and surrounding areas through monoamine reuptake [1]. Cocaine inhibition of these reuptake processes results in prolonged monoamine elevation in brain regions that promote reward and addiction [1]. Cocaine also blocks sodium channels but with lower potencies (50 µM or higher) [5]. These cocaine targets are expressed in both the CNS and the peripheral systems [6], [7].

Many chemical analogs of cocaine have previously been synthesized [8], [9]. Cocaine methiodide (CM) is a chemical analog of cocaine with a stable positive charge at physiological pH. The positive charge of CM prevents a systemic administered dose from crossing the blood brain barrier [10]. Therefore, CM should only inhibit the functions of cocaine target proteins in peripheral tissues.

It has been observed that the toxic effects of systemic CM, measured in vivo by median lethal doses (LD_50_), are similar to that of cocaine [11], [12], leading to the presumption that the potencies of CM and cocaine for peripheral targets might be similar. Accordingly, several investigations examined the effects of systemic CM with the presumption that the results represented cocaine interactions with peripheral cocaine targets at similar doses [10], [13].

However, some studies have shown that CM and cocaine may have different potencies at cocaine targets. CM was shown to be less potent than cocaine at inhibiting NE uptake in aortic tissues dissected from guinea pigs and rats [14]. CM was found to be less potent than cocaine at inhibiting the binding of mazindol [15] to rat striatal tissue preparations. In addition, in vivo data showed that CM via intracranial delivery did not produce comparable results to cocaine in rat self-administration tests [16].

While these prior CM studies are relevant to compare the effects of CM to cocaine, they were performed in tissue preparations that contain multiple cocaine targets with varying expression levels. Accordingly, the concentration-responses for these two drugs have not been clearly determined for each major target of cocaine (DAT, NET, SERT or sodium channel subtypes). Therefore, we aimed to determine the potency of CM and cocaine at inhibiting major cocaine target proteins and thus testing the hypothesis that CM is similarly potent as cocaine and would produce similar effects in peripheral tissues.

## Results

**Figure 1:**
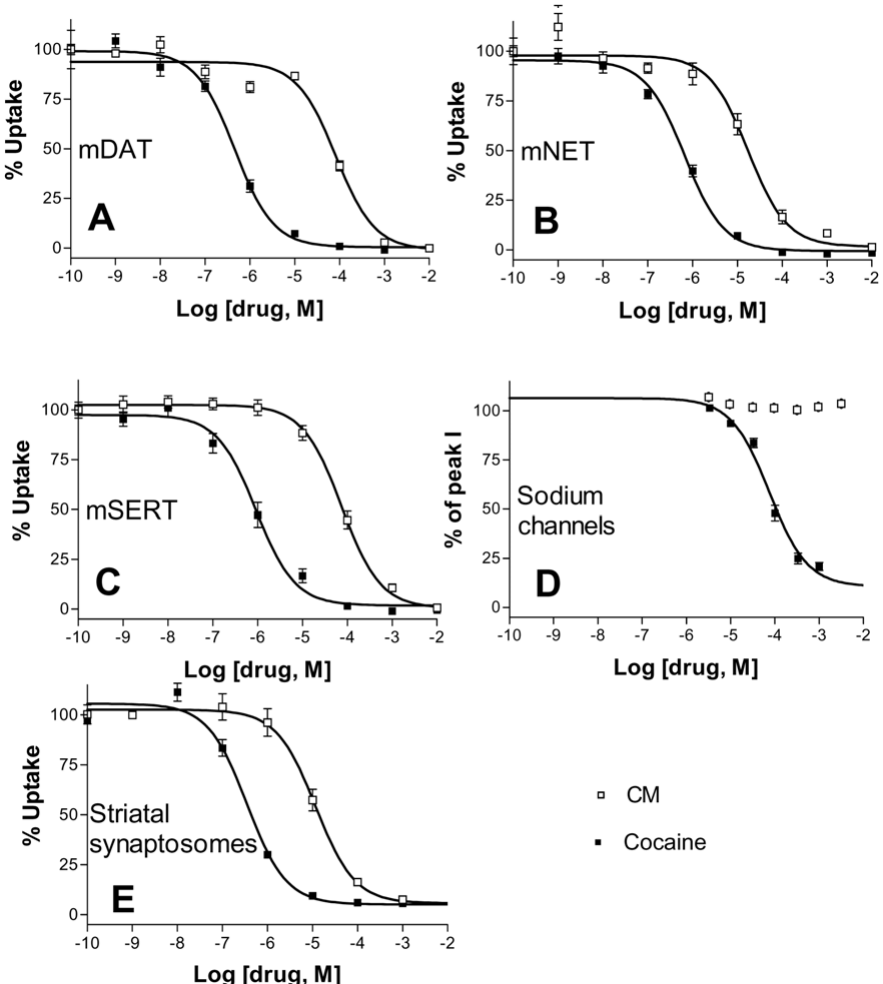
Concentration-response curves for cocaine and cocaine methiodide. Dopamine uptake by mouse DAT (A), norepinephrine uptake by mouse NET (B), and serotonin uptake by mouse SERT (C) into transfected cells were measured in the presence of increasing concentrations of cocaine or cocaine methiodide. The Na^+^ channel currents in isolated mouse DRG neurons were recorded by whole-cell patch clamping (D). The data are presented as the percent of the pre-drug activity. Dopamine uptake by striatal synaptosomes were measured in the presence of increasing concentrations of cocaine or cocaine methiodide (E). For A, B, C, and E, each data point represents the average of triplicate measurements ± standard error of means and the experiments were repeated three times with similar results. For D, each data point was obtained from four cells.

Previous pharmacological studies of CM on CNS proteins utilized dissected tissues or tissue homogenates. Depending on the source, these homogenized tissue samples have variable expressions of multiple cocaine target proteins. In addition, monoamine transporters share substrates and high affinity inhibitor compounds (such as mazindol) commonly used to study drug binding to the transporters. To study the effect of CM on individual transporter cocaine targets, we used cells transiently transfected with individual transporter cDNAs. Because mouse models were used in recent publications on the study of CM [7], [17], [18] we chose the three mouse monoamine transporter cDNAs for transfection. Figures 1A–C show the concentration-response curves for CM and cocaine inhibition of monoamine uptake by each of the three transporters. Each experiment was run in triplicate and the experiments were repeated 3 times with similar results. The average IC_50_ values for cocaine and CM respectively were: for mDAT, 0.45±0.11 µM and 83.2±2.1 µM, a 184 fold increase; for mNET, 0.67±0.09 µM and 20.9±3.1 µM, a 31 fold difference; and for mSERT, 0.68±0.39 µM and 84.3±4.8 µM, a 123 fold difference. Student's paired t-tests showed that the IC_50_ values of CM and cocaine were significantly different for each of the 3 transporters (p<0.001 for all three comparisons). These results are summarized in Table 1.

**Table 1:** Cocaine and cocaine methiodide IC_50_ values for the inhibition of monoamine transporters and DRG neuron Na^+^ channels.

IC50 [µM]^a^	Cocaine	Cocaine Methiodide	ratio	p value^b^
Striatal synaptosomes	0.35±0.11	11.5±5.1	33	<0.001
mDAT	0.45±0.11	83.2±2.1	184	<0.001
mNET	0.67±0.09	20.9±3.1	31	<0.001
mSERT	0.68±0.39	84.3±4.8	123	<0.001
DRG Na+ Channels	84.8±5.9	not measured	---	---

a The IC_50_ values are mean ± standard error of means calculated from 3 independent experiments.

b Significance was determined by Student's paired t-test.

In addition to the experiments with cultured cells expressing the transporters, we also examined CM and cocaine inhibition of dopamine uptake in mouse brain tissues. Synaptosomes were prepared from the striatum of healthy adult C57B6 mice. Standard uptake assays were performed to measure CM and cocaine inhibition. The results are shown in Figure 1E (n = 3, p<0.001). The average IC_50_ values for cocaine and CM were 0.35±0.11 µM and 11.7±5.1 µM respectively, a difference of 33 fold.

Cocaine is well known to block the function of sodium channels [19]. However, the effect of CM on sodium channels has not been reported. Since CM is primarily used to identify cocaine effects in the periphery, where sodium channels are potential targets, we sought to determine whether CM and cocaine had similar potencies on sodium channels in peripheral nerves. DRG neurons express several different fast and slow responding sodium channel subtypes, including Na_V_1.1, Na_V_1.6, Na_V_1.7, Na_V_1.8, and Na_V_1.9 [20]. DRG neurons provide convenient samples to examine the inhibitory effect of cocaine and CM on multiple sodium channels.

**Figure 2:**
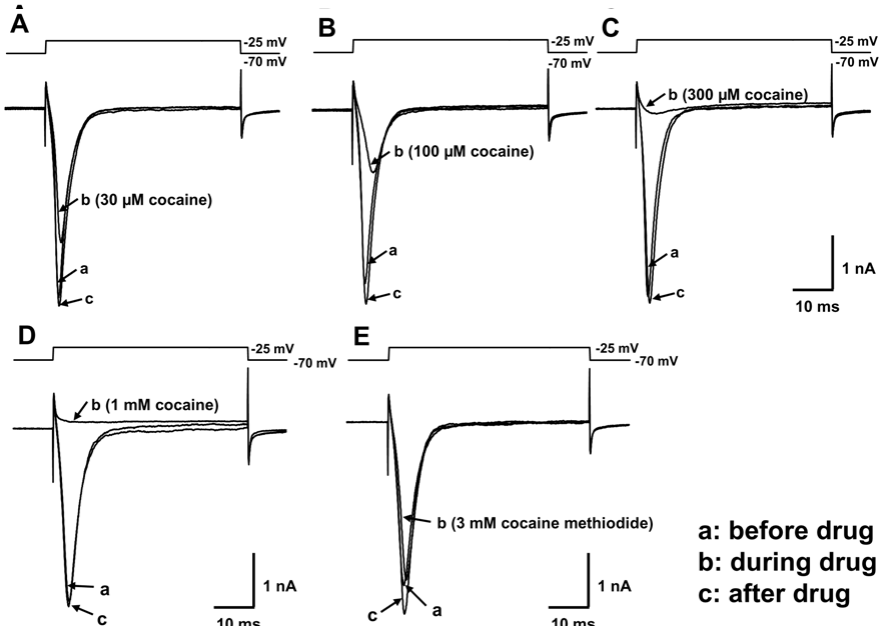
Effects of cocaine and cocaine methiodide on sodium channels in DRG neurons. Isolated mouse DRG neurons were voltage-clamped in the whole-cell mode, held at −70 mV and sodium channels were activated by 10 consecutive pulse stimulations (10 Hz, 50 ms duration, depolarized from −70 mV to −25 mV). Representative currents recorded before drug addition (before drug), during the drug (during drug) and after drug washout (after drug) are overlaid for 30 (A), 100 (B), 300 (C) and 1,000 µM (D) cocaine as well as 3 mM cocaine methiodide (E).

Whole-cell recordings were performed using mouse DRG neurons. Sodium currents were elicited by depolarization to desired test potentials from the holding of −70 mV in the absence and presence of cocaine or CM as described in Materials and Methods. Figure 2 shows representative inward current traces recorded from DRG neurons, before and during cocaine application, as well as after cocaine washout. As shown in Figure 2A–2D, cocaine dose dependently inhibited the rising phase of the inward current. Peak currents obtained from 10 voltage pulses were averaged for each drug concentration and normalized to the control value before the drug application. The concentration-response curve is shown in Figure 1D for comparison with the monoamine transporters. IC_50_ values were determined for cocaine inhibition of the sodium channels in mouse DRG neurons with an average value of 84.8±5.9 µM (n = 4) (Table 1). This data is consistent with previous work that the potency of cocaine in inhibiting sodium channels is roughly 100 fold lower than those for the monoamine transporters [5].

However, when CM was applied to isolated DRG neurons, little effect was observed on the sodium channel currents. Even at 3 mM of CM, the highest concentration tested, very little inhibition was observed in peak inward current (Figure 2E, n = 4 cells, p>0.05) and the data did not allow the calculation of IC_50_ value. While concentrations higher than 3 mM may inhibit sodium channels, doses equivalent to such concentrations would be much higher than the LD_50_ and thus not likely to be used in animals. Therefore, we limited our experiments to 3 mM. Figure 1D shows the effect of CM on sodium channel currents from 3 µM to 3 mM. To rule out that the ECS used in the DRG experiments may interfere with CM actions, we performed transport assays with HeLa cells using the ECS as for the DRG experiments. The results were not different from the experiments using the PBS/Ca/Mg buffer (data not shown).

## Discussion

Since CM cannot pass across the blood brain barrier, it had been used in studies attempting to separate the CNS effects from the peripheral effects of cocaine. The effective concentrations for CM and cocaine used in these studies were similar based on the assumption that the IC_50_ values for CM and cocaine are similar for major target proteins. The data presented in this paper shows very substantial differences between potencies of cocaine and CM in inhibition of major cocaine target proteins.

We found that the individual IC_50_ values for CM are 184-fold, 31-fold, and 123-fold higher than those for cocaine at DAT, NET, and SERT respectively. We also found that cocaine inhibits the sodium channels in DRG neurons with an apparent IC_50_ of 85 µM, about 100-fold higher than those for the monoamine transporters. The sodium channel inhibition data shown in Figure 1D fit well with a single IC_50_ value, suggesting that the individual CM IC_50_ values for each sodium channel subtypes are similar. In contrast, doses of CM up to 3 mM have little observable effect on sodium channels expressed in DRG neurons. It remains to be determined whether the sodium channel subtypes that are not expressed in DRG neurons can be inhibited by CM and whether those channels have similar sensitivities to CM and cocaine.

Cocaine has been referred as a “dirty drug” due to its multiple sites of action within the CNS and peripheral tissues [21]. Separating the cocaine actions in the CNS from peripheral tissue contributions can be beneficial in understanding complex cocaine effects. Our data show that CM and cocaine have very different potencies on the major cocaine targets, and therefore, equimolar systemic doses of CM do not produce equivalent inhibition of major cocaine targets.

Our data with DRG neurons raises interesting questions about how systemic CM produces its effects which was hypothesized to result primarily from CM inhibition of peripheral Na^+^ channels [7]. Our CM electrophysiology data excludes the five sodium channel subtypes commonly expressed in DRG neurons from mediating this peripheral effect. The effects of CM at other sodium channel subtypes, not expressed in DRG neurons, remain unknown. Indeed, recent reports show that the peripheral glutamate system is involved in rapid CNS effects observed with intravenous administration of cocaine and CM [18]. However, it is clear from our data that equivalent doses of CM should not be used to examine the effects of cocaine interaction with its peripheral targets.

The use of CM to measure the effects of peripheral cocaine stems from previous reports of similar LD_50_ values for cocaine and CM [11], [12], which suggests the two drugs have similar potencies at the targets that mediate the lethal effects. In stark contrast, we observed remarkable differences between the potencies of CM and cocaine. Our results suggest that the lethal toxic effects of cocaine and CM are not likely through the inhibition of the monoamine transporters or subtypes of sodium channels expressed in DRG neurons. Other target proteins might be involved. One study shows that cocaine is a low affinity antagonist at α7-nicotinic acetylcholine receptor (nAChR) while CM is a high affinity agonist of the receptor [22]. Another study indicates that CM produces weaker effect than cocaine in a conditioned taste aversion test [23]. It has also been proposed that the toxic effects of cocaine emanate from an unknown site in the peripheral tissue [6]. Future experiments are needed to identify other CM target proteins and to understand why CM and cocaine have similar LD_50_ in animals.

The data presented here show that CM is much less potent than cocaine at inhibiting monoamine transporters and thus similar doses of CM will not inhibit the transporters to the same extent. We also observed that a very high dose of CM does not inhibit sodium channels expressed in DRG neurons. Therefore, systemic CM effects are not good measurements of cocaine actions through its peripheral targets.

## Materials and Methods

### Substrate reuptake into transiently transfected cells

Plasmid DNA containing mDAT, mNET, and mSERT were cloned into bluescript vector with a T7 promoter as described [24], [25]. HeLa cells (American Type Culture Collection, Rockville, MD) were grown in 96-well plates, infected with recombinant vTF-7 vaccinia virus, carrying the T7 polymerase gene, and transiently transfected with the plasmids bearing cDNAs using Lipofectin (Invitrogen Corp., Carlsbad, CA) as described previously [24].

About 20–24 h after transfection, HeLa cells were assayed for substrate uptake in 96-well plates at room temperature using the PBS/Ca/Mg buffer (phosphate buffered saline solution containing 1 mM MgCl_2_, 0.1 mM CaCl_2_, and 50((M L-ascorbic acid). For determination of IC50 values, cells were co-incubated in the PBS/Ca/Mg buffer with added 60 nM [3H]-labeled monoamine substrates and increasing concentrations of an inhibitor (e.g., cocaine or CM). Uptakes were terminated by two successive washes with PBS/Ca/Mg. Amounts of [3H]-labeled substrates accumulated in the cells were quantitated by liquid-scintillation counting. All experiments were performed in triplicates. Cells transfected with vehicle were used as controls and radioactivity associated with these cells were considered the background. This background was subtracted from the total scintillation counts of the wells.

### Dopamine reuptake into synaptosomes

All animal work was conducted in adherence to OSU IACUC approved protocols and guidelines for animal welfare. C57B6 mice (aged 6–8 weeks) were decapitated and striatum were dissected from both sides of the brain and stored on ice. The tissues were placed in ice-cold Krebs'-Ringer's solution buffer (KRB) (in mM: 125 NaCl, 1.2 KCl, 1.2 MgSO4, 1.2 CaCl2, 22 NaHCO3, 1 NaH2PO4, and 10 glucose, adjusted to pH 7.4) with an additional 0.32 M sucrose. Tissue samples were homogenized by using a glass homogenizing tube and with a Teflon-coated pestle. The samples were centrifuged for 10 min at 1,000xg. Supernatant was collected and the debris pellet was discarded. Supernatant was centrifuged for 15 min at 16,000x g. The resulting pellet contained synaptosomes, and was resuspended in KRB supplemented with pargyline (50((M) and ascorbic acid (100 µM). Synaptosomes were assayed for substrate uptake at room temperature using a PBS/Ca/Mg buffer (phosphate buffered saline solution containing 1 mM MgCl_2_, 0.1 mM CaCl_2_, and 50 µM L-ascorbic acid). For determination of IC_50_ values, synaptosomes were co-incubated in the PBS/Ca/Mg buffer with added 60 nM [^3^H]-labeled dopamine, 100 µM desipramine (NET-selective inhibitor) and increasing concentrations of an inhibitor (e.g., cocaine or CM). Uptakes were terminated by two successive washes with PBS/Ca/Mg and the vesicles containing the transported substrates were collected through 96-well microfilter plates (Millipore, Irvine, CA). Amounts of [^3^H]-labeled dopamine accumulated were quantitated by liquid-scintillation counting. All experiments were performed in triplicates. Synaptosomes with the highest dose of inhibitor were used as background controls and radioactivity associated with these wells were subtracted from the total scintillation counts of all wells.

### Whole-cell voltage clamp recording of sodium channel currents in dorsal root ganglion neurons

Dorsal root ganglion (DRG) neurons (T1–T10) from adult male C57B6 mice (aged 6–8 weeks) were isolated as per Malin et al. [26] Briefly, ganglia were dissected under stereo microscope and washed in Ca^2+^/Mg^2+^-free Hank's Buffered Salt Solution (HBSS). DRGs were digested enzymatically, first with papain and then collagenase II and dispase II, each for 10 min at 37°C. Digested DRGs were then triturated in culture media (F-12 supplemented with 10% FBS and 5 mg/ml penicillin/streptomycin) by a fire-polished Pasteur pipette until solution becomes cloudy. Isolated DRG neurons were plated on poly-ornithine coated glass cover slips and maintained in a 37°C, 5% CO_2_ incubator. Individual DRG neurons were recorded after overnight culture.

Plated coverslips were centered in a perfusion chamber filled with extracellular solution (ECS) containing (in mM): 140 NaCl, 5 KCl, 2 CaCl_2_, 1 MgCl_2_, 10 glucose, 10 HEPES, pH 7.4. Cocaine and CM were dissolved in ECS and delivered via a pressure-driven perfusion system (SmartSquirt 8, AutoMate Scientific) with the tip positioned so the DRG neuron being recorded was fully within the direct stream of perfusate. Recording pipettes were pulled from micropipette glass (World Precision Instruments, Sarasota FL) to 2–4 MΩ when filled with an intracellular solution containing (in mM): 140 CsCl_2_, 1 CaCl_2_, 2 MgCl_2_, 11 EGTA, 10 HEPES, 2 Mg_2_ATP, pH 7.2). Whole-cell recordings were made using an EPC10 amplifier and the PatchMaster 2.2.0 software (both from HEKA Electronik, Germany). As soon as the whole-cell configuration was established, fast and slow capacitances were cancelled and the holding potential (Vh) was set to -70 mV. A step protocol (16 steps from −70 mV to 10 mV with 5 mV increment for each step) was applied to determine the testing voltage (Vt) that generated the maximal inward current in the following experiment. Sodium channels were activated by a 10 consecutive pulse stimulation (10 Hz, 50 ms duration, depolarized from Vh to Vt). Data were filtered at 3 kHz and digitized at 20 kHz. All recordings were made at room temperature (22–24°C). Only one cell per cover slip was recorded to avoid possible drug contamination of other cells. Representative current traces were redrawn in Origin 8.0 SR1 (Northampton, MA).

A range of drug concentrations, starting from low to high, were tested for each DRG neuron. The sodium channel currents were recorded 30 s before the drug application, during the 30 s drug application, and 30–60 s after drug washout by perfusion with ECS. For each set of tests, current amplitude was measured by subtracting the baseline value from the peak current. The current amplitude during the drug was normalized to that before the drug application, and the normalized values were used to plot the dose-response curve. The currents measured after the drug washout were used to confirm the complete recovery before testing the next drug concentration.

### Data analysis

The IC_50_ values were determined by nonlinear regression analyses of experimental data using GraphPad Prism 3.0 (San Diego, CA). IC_50_ values presented are averages ± standard error of means (SEM) calculated from 3 independent uptake experiments or recordings of 4 different DRG neurons. Statistical analyses for the difference between the IC_50_ values of the two drugs were performed by Student's paired t-test using SPSS 17.0 (Chicago, IL).
